# Geographic Variations of Stroke Hospitalization across France: A Diachronic Cluster Analysis

**DOI:** 10.1155/2018/1897569

**Published:** 2018-07-18

**Authors:** Yacine Lachkhem, Étienne Minvielle, Stéphane Rican

**Affiliations:** ^1^Equipe d'Accueil Management des Organisations de Santé, French School of Public Health, avenue du Professeur Léon-Bernard, 35043 Rennes, France; ^2^Gustave Roussy Cancer Center, 114 rue Edouard Vaillant, 94805 Villejuif, France; ^3^LADYSS, Paris Nanterre University, F92000 Nanterre, France

## Abstract

**Background:**

This study evaluates the clustering of hospitalization rates for stroke and compares this clustering with two different time intervals 2009-2010 and 2012-2013, corresponding to the beginning of the French National Stroke Plan 2010–2014. In addition, these data will be compared with the deployment of stroke units as well as socioeconomic and healthcare characteristics at zip code level.

**Methods:**

We used the PMSI data from 2009 to 2013, which lists all hospitalizations for stroke between 2009 and 2013, identified on the most detailed geographic scale allowed by this database. We identify statistically significant clusters with high or low rates in the zip code level using the Getis-Ord statistics. Each of the significant clusters is monitored over time and evaluated according to the nearest stroke unit distance and the socioeconomic profile.

**Results:**

We identified clusters of low and high rate of stroke hospitalization (23.7% of all geographic codes). Most of these clusters are maintained over time (81%) but we also observed clusters in transition. Geographic codes with persistent high rates of stroke hospitalizations were mainly rural (78% versus 17%,* P *< .0001) and had a least favorable socioeconomic and healthcare profile.

**Conclusion:**

Our study reveals that high-stroke hospitalization rates cluster remains the same during our study period. While access to the stroke unit has increased overall, it remains low for these clusters. The socioeconomic and healthcare profile of these clusters are poor but variations were observed. These results are valuable tools to implement more targeted strategies to improve stroke care accessibility and reduce geographic disparities.

## 1. Introduction

Stroke is a leading cause of death, adult long-term disability, and loss of quality-adjusted life years in western countries [1]. It is a major public health issue with an increasing economic and social burden given the aging population context (the risk of stroke increases with age) and the potentially devastating consequences for survivors [2]. Stroke management requires neurology expertise and specific resources in a restrictive timeframe.

Geographic variations of stroke risk have been revealed at various scales. In the US, the so-called “stroke belt ”, has been monitored since 1939 [3]. Across Europe, an east-west decreasing gradient has been observed by compiling multiple stroke registries [4]. In France, recent studies reported association between increasing neighborhood deprivation and stroke mortality [5, 6]. Because access to stroke care can explain a part of these variations, the French ministry of health launched the Stroke National Action Plan, 2010–2014. One of the objectives of this plan was to increase the deployment of stroke units in order to constitute a regional stroke care network with these units at the center of the care pathway.

Various studies focused on the identification of the barriers influencing stroke care and showed a wide spectrum of factors, related to the patient (stroke knowledge, age, stroke severity, etc.) [7] and to the spatial (deprivation areas, distance, etc.) [8] or organizational factors (transport modality, prenotification, etc.) [9] or associated with physicians training [10]. In this context, interventions to improve care pathways have, on the one hand, pushed towards a global approach by supporting the implementation of combined interventions that cover all the acute care pathway [11]. On the other hand, to increase efficiency, these interventions must also be targeted. Spatial statistics methods prove to be valuable tools in a public health approach aimed at identifying risk areas where it appears relevant to strengthen, adapt, or prioritize action.

We used zip code level data and spatial statistics to identify geographic clusters of high and low rate of stroke hospitalization from 2009–2010 to 2012–2013 in a nationwide analysis. We them compared to stroke unit spreading to assess accessibility and used socioeconomic, healthcare, and environmental data to characterize identified clusters.

## 2. Method

### 2.1. Study Population

The main variable of our study is the stroke hospitalization rate. We extracted all hospitalizations for stroke from the national PMSI database. The PMSI (Programme de Médicalisation des Systèmes d'Information) is the French Medical Information System provided by the ATIH (Technical Agency for Information on Hospitalization) which covers all French public and private hospitals. Stroke hospitalization was selected using the ICD-10 code (10th revision of the International Statistical Classification of Diseases and Related Health Problems) referring to cerebrovascular disease (I60, I61, I62, I63, I64, and G46) recorded as a principal discharge diagnosis. These data have been extracted for two periods: 2009-2010 and 2012-2013 and restricted to France Metropolitan Area. All hospitalizations were assigned to PMSI geographic codes available in the database which indicate where the patient lives and correspond to ZIP codes. Hospital stays ending and stroke unit admission were also extracted. Two-year stroke hospitalization rates were calculated for each period and standardized on age and sex using the 2011 French census population as reference and we used the Empirical-Bayes standardization method to account for the variance instability caused by small population size.

Healthcare, environmental, and socioeconomic variables were collected at the PMSI code level. Healthcare profile is described by the distance between the patient and the location of its hospital admission, calculated using OSRM engine (Open Source Routing Machine) and GP (General Practitioner) accessibility and availability using the LPA indicator of 2010 and 2013. The Local Potential Accessibility (LPA) is a local indicator of accessibility applied to GP that also considers healthcare supply and demand factors in neighboring municipalities. Urban/rural profiles were collected from 2010 INSEE (Institut National de la Statistique et des Etudes Economiques: The French National Institute for Statistics and Economic Studies) two-level typology (urban/rural and city center, suburb, isolated city, and rural areas). Finally, the socioeconomic profile was estimated using the FDep deprivation index that considers four variables: average household income, percentage of high school graduates in the population aged ≤15 years, percentage of blue-collar workers in the active population, and unemployment rate [12].

### 2.2. Spatial Clustering Analysis

To understand where on the landscape clustering of stroke hospitalization rates may be occurring, Getis-Ord Gi*∗* statistics were used [13]. The clustering was assessed at national scale. We used the Gi*∗* statistics at France Metropolitan Area scale to identify the location of significant hotspots/coldspots clusters of stroke hospitalization rate using first-order queen contiguity spatial weight and nine hundred ninety-nine Monte Carlo replications at *P* value less than 0.05. Hotspots represent higher hospitalization rates than would be expected if distribution was random, while coldspots represent lower hospitalization rates than would be expected if distribution was random. In terms of temporal trends, we follow the approach of Schieb et al. and compare Gi*∗* for 2009–2010 with clusters for 2012–2013 by categorizing them as persistent and transitional clusters [14].

All spatial clustering analysis was implemented in GeoDA software, statistical analysis was performed using JMP 13 (SAS Institute Inc., Cary, NC), and mapping was performed in ArcGIS 10.5 (Esri). According to French governmental regulations, the examination of the study protocol by the National Ethics Committee was not required.

## 3. Results

Our two study periods represent 405,874 stroke hospitalizations. The age-sex-adjusted stroke hospitalization rate was 169 per 100,000 during 2009–2010 and increased to 179 per 100,000 during 2012–2013. Cluster analysis conducted at the national scale using the Gi*∗* statistic identified areas where the stoke hospitalization rate was statistically higher. These high rate clusters represent 5.3% of all geographic codes (n = 303) and were mainly located along a northeast/southwest axis and sparkled in various regions such as Bretagne, Rhône-Alpes, Nord-Pas-de-Calais, and southwest regions.

18.4% of all geographic codes are low rate clusters and were mainly observed in the northwest region such as Haute-Normandie, Île-de-France, a large part of the Center region, and Pays-de-la-Loire. We also observed these clusters along the Mediterranean coast and in the Rhône-Alpes region. In both cases, we find a similar distribution over the previous study period.

**Figure 1:**
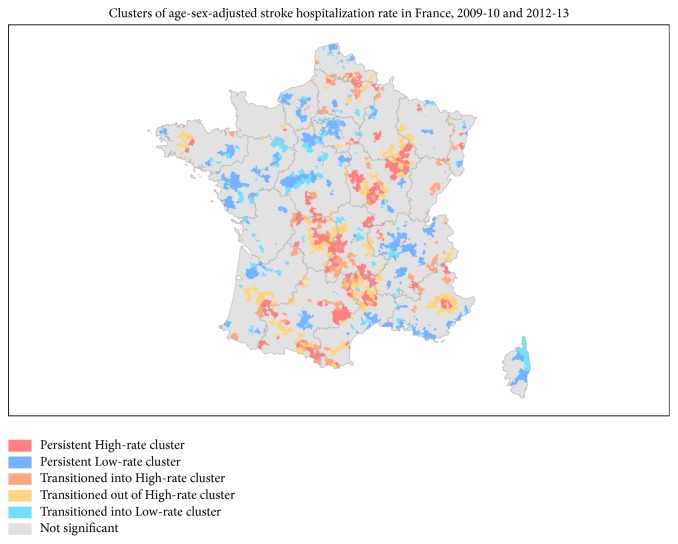
Clusters of age-sex-adjusted stroke hospitalization rate in France, 2009-2010 and 2012-2013.

Regarding the comparison between Gi*∗* clusters 2009–2010 and 2012–2013, 48% of high rate clusters remained over the two-study period (n = 177) with the same spatial pattern despite a decreasing area (see Figure 1). If the northeast/southwest axis remained, regional singularities identified earlier are attenuated. Regarding low rate clusters, 81% of them identified in 2009–2010 remained in 2012–2013 (n = 841) with a reinforcement of the axis from the Île-de-France region to the Pays-de-la-Loire regions. Geographic code that transitioned into high rate clusters was mainly located along the northeast/southwest axis and close to high rate clusters in Nord-Pas-de-Calais, Picardie, or the southwest. We also identified geographic codes that transitioned out of high rate clusters. It represents 14% of the statistically significant clusters and shared a similar spatial pattern.

Healthcare and socioeconomic profiles of geographic codes can be compared in Table 1. In general, geographic codes located in a persistent high rate cluster had a disadvantageous healthcare and socioeconomic profile compared to geographic codes identified in a persistent low rate clusters. Mainly located in a rural area, high rate cluster was described with longest admission travel (42.4 min versus 29.3 min,* P* <.0001) and lower accessibility to stroke units (29.5% admissions versus 40.5%,* P* <.0001). In-hospital mortality was significantly higher (15.3%,* P* <.0001) and only accessibility to the GP was similar. In terms of socioeconomic conditions, persistent high rate cluster reported a higher rate of disadvantaged geographic codes (31.1% versus 11.1%,* P* <.0001). Regarding transitioned clusters, we observe a similar trend between geographic codes that transitioned into or out of a high rate cluster and a persistent high rate cluster. Geographic codes that transitioned into a low rate cluster were described as a hybrid profile. Geographic codes that transition into a high rate cluster had better accessibility to stroke units where geographic codes that transitioned out of a high rate cluster were more distant. Geographic codes that transitioned into a low rate cluster were described by an urban-rural balance and a distance and accessibility to the average between high and low rate cluster.

## 4. Discussion

Our study reveals that high rate geographic codes (N= 177) were mainly located in a northeast/southwest axis and small clusters are sparkled at regional and interregional scales. Low rate geographic codes (N= 841) were observed in an axis between Île-de-France and Pays-de-la-Loire region and in a second axis between Rhône-Alpes and Bouche du Rhône region. The observed geographic code remains stable between the two study periods. However, we observed some variations: 126 geographic codes transitioned into high rate clusters, 189 transitioned out of high rate clusters, and 200 transitioned into low rate clusters.

Geographic codes with persistent high rates of stroke hospitalizations were mainly rural and had a least favorable socioeconomic and healthcare profile. These findings are consistent with previous studies assessing such relationships. Associations between neighborhood deprivation and stroke have been reported across the entire stroke care pathway. In Japan, a large population based prospective study of 91,723 men and women aged 40–69 years reported the impact of neighborhood deprivation level (i.e., the proportions of elderly couple households, elderly single households, single-mother households, sales and service workers, agricultural workers, blue-collar workers, and unemployed persons) on stroke incidence even after adjustment for individual socioeconomic indicators [15]. A Danish study observed reduced odds of having good-quality acute treatment and increased mortality for low socioeconomic status patients (defined as income, education, or occupation) [16].

In our study, healthcare situation has been observed using both GP and stroke unit accessibility. Our findings revealed no association with GP access. However, high rate clusters were mainly located in rural areas with longer distance and poorer admission rate to stroke unit. These results are consistent with studies exploring geographic disparities of stroke hospitalization. In Florida, excess risks of stroke hospitalization and mortality have been located in north, more rural compared to the rest of the state [17]. Difference has also been observed in stroke management and poststroke rehabilitation [18, 19]. These works revealed suboptimal care provided in rural area with significant care quality disparities.

During our study period, 43 stroke units were created in France. Globally, distance to stroke reduces from 45.5 min to 36.6 min and stroke unit admission increased from 23.3% to 40.4%. However, for persistent high rate clusters, stroke unit accessibility did not reduce significantly. While rural-urban health disparities are widely recognized and documented, our study identified two points to highlight. First, issues observed in rural areas are complex and cannot be addressed in a standardized way. Observed cumulative disadvantages differ according to the areas analyzed indicating heterogeneous weight of associated factors. Second, stroke unit accessibility for areas with low-populated areas raises the question of the cost/benefit of major stroke unit deployment. Even if at patient-level, the benefit would be clear, the heavy resource investment compared to the small number of patients who benefit from it remains expensive for the health system, forcing us to explore specific strategies for these areas.

In France, a telestroke network has been tested in three pilot regions according to the Stroke Plan 2010–2014 and appeared to be effective and safe with an increase of the proportion of patients who will access thrombolysis [20]. It is consistent with longer experienced telestroke network which showed an increase of access to stroke unit and thrombolysis rates and associated with long-term improvement in terms of quality indicators of acute hospital care [21]. Complementary thinking should also be conducted on organizational change. Implementation of a hub and spoke model provides successful evidence for improving stroke care in rural locations. In New South Wales (Australia), a historical control cohort study reports improved stroke care in rural hospital with increase of patients discharged home after the implementation of a hub and spoke model driven by clinical coordinators [22].

This study is not without limitations. First, it is a nationwide study using administrative data. Parameters applied for the analysis at this scale do not allow us to report on variations appearing at regional scale. Because of the absence of distinction between first-ever and recurrent stroke on the database, we used the algorithm of patient selection already used by other French studies using the national PMSI database [23]. Second, if geographic codes can mostly be referred to as zip codes, municipalities under 1000 inhabitants are aggregated according to the anonymity constraints.

This study identified persistent high rate stroke hospitalization clusters with their socioeconomic and healthcare characteristics helping to prioritize interventions. The spatiotemporal approach is also relevant because it attempts to highlight dynamics by identifying geographic codes that transitioned into high or low rate clusters, regarding stroke unit deployment efforts. Several studies use spatial statistics method in stroke but also in breast cancer [24], lower respiratory tract infection [25], or suicide [26] and make it an essential decision-making tool to implement effective strategies to improve health and reduce geographic disparities.

In France, geographic disparities of stroke hospitalization exist. Stroke high rate hospitalization clusters were mainly located in rural areas, with longer distance to stroke unit and low socioeconomic characteristics. To attain these areas, new strategies should be adopted that consider these isolated areas and associated factors. Several works observed the persistent disadvantage of rural areas, explained by the combination of lack of healthcare facilities and demographic/socioeconomic factors [27, 28]. Implementation of telemedicine strategy to bypass the distance threshold and organized stroke care to improve the efficacy of available resources are strategic. But because stroke factors are complex, global approach became fundamental and should be context-based, targeting public prevention with various vectors and mobilizing all parts of the stroke care chain.

## Figures and Tables

**Table 1:** Comparison of persistent high-rate clusters with persistent low-rate clusters and transitional clusters.

		*Persistent clusters*			*Transitioned clusters*					
		High-rate	Low-rate	*P *value	Into High-rate	*P *value	Out of High-rate	*P *value	Into Low-rate	*P *value
	Number of clusters	177	841		126		189		200	
	Number of patients, 2009/10	3850	56978	<,0001	2921	<,0001	3900	<,0001	8103	<,0001
	Number of patients, 2012/13	4042	61433	<,0001	3111	<,0001	4019	<,0001	8068	<,0001
Urban/Rural	Rural	78%	17%	<,0001	77%	0,7409	83%	0,1854	44,2%	<,0001
	Urban	22%	83%	<,0001	23%	0,7409	17%	0,1854	55,8%	<,0001
Among urban unit	City center	7%	7%	0,7123	7%	0,9383	4%	0,1949	7,6%	0,8393
	Suburb	10%	70%	<,0001	13%	0,5279	9%	0,7430	42,1%	<,0001
	Isolated city	4%	6%	0,2051	3%	0,5641	4%	0,8287	6,1%	0,3886
Healthcare	Admission to SU	29,5%	40,5%	<,0001	35,3%	0,0281	30,3%	0,7554	35,9	0,0061
	Nearest SU (min)	55,3	17,1	<,0001	49,1	<,0001	61,1	<,0001	31,6	<,0001
	Admission distance (min)	42,4	29,3	<,0001	41,0	0,6572	48,8	0,0217	34,4	0,0035
Stay output	Home	46,4%	53,0%	<,0001	51,5%	0,0019	47,4%	0,5113	51,0%	0,0015
	Death	15,3%	13,9%	<,0001	15,4%	0,9269	17,4%	0,0361	16,1%	0,4009
	Mutation	10,7%	8,2%	0,0017	8,9%	0,1036	10,0%	0,5094	9,4%	0,1790
	Transfer	27,6%	24,9%	0,0105	24,3%	0,0270	25,2%	0,0767	23,5%	0,0027
	GP accessibility 2013	48,9	62,7	<,0001	47,9	0,6575	45,9	0,1710	62,2	0,0256
Socioeconomic	FDep index	31,1%	11,1%	<,0001	30,2%	0,0490	26,5%	<,0001	14,0%	<,0001

## Data Availability

The data used to support the findings of this study are available from the corresponding author upon request.
